# Supplementary material for: The Evolution and Characterization of the RNA Interference Pathways in Lophotrochozoa
Source: Genome Biol Evol. 2024 May 7;16(5):evae098. doi: 10.1093/gbe/evae098 (PMC11114477; doi:10.1093/gbe/evae098)
Supplement: evae098_Supplementary_Data [file evae098_supplementary_data.zip › Supplementary_materials.zip!/selected_miRNAs/NoGe_selected_miRNAs/novel_miRNA_families_pdf_files/NMRB01002238.1_Notospermus_geniculatus_strain_Ushimado_scaffold2238,_whole_genome_shotgun_sequence_36957.pdf]

Provisional ID	:	NMRB01002238.1_Notospermus_geniculatus_strain_Ushimado_scaffold2238,_whole_genome_shotgun_sequence_36957
Score total	:	846
Score for star read(s)	:	3.9
Score for read counts	:	840.8
Score for mfe	:	1.9
Score for randfold	:	0
Score for cons. seed	:	-0.6
Total read count	:	1661
Mature read count	:	1649
Loop read count	:	0
Star read count	:	12

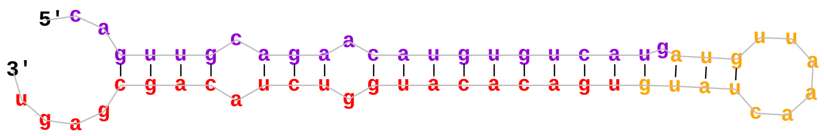

Genome coordinates: NMRB01002238.1 11117-11174

pre-miRNA sequence:
CAGTTGCAGAACATGTGTCATGCAGTTGCAGAACATGTGTCATGATGTTAAACTATGTGACACATGGTCTACAGCGAGT

Mature sequence: TGACACATGGTCTACAGCGAGT

BLAST results for the pre-miRNA sequence against the *Lineus longissimus* genome assembly:

Scaffold	Start	Stop	Identity	e-value
OU342992.1 1	6582057	6581998	88.333	1.60e-11

```
Nge_novel_miRNA     CAGTTGCAGAACATGTGTCATGATGT--TA-AACTATGTGACACATGGTCTACAGCGAGT
Llo_genome          CAGTTGCAGAACGTGTGTCATAATGTCATATAATCATGTGACACATGGTCTACAGCGAGT
```
